# Boosting Neurogenesis in the Adult Hippocampus Using Antidepressants and Mesenchymal Stem Cells

**DOI:** 10.3390/cells11203234

**Published:** 2022-10-14

**Authors:** Marta Kot, Pawan Kumar Neglur, Anna Pietraszewska, Leonora Buzanska

**Affiliations:** Department of Stem Cell Bioengineering, Mossakowski Medical Research Institute, Polish Academy of Sciences, Pawinskiego 5 Street, 02-106 Warsaw, Poland

**Keywords:** adult hippocampal neurogenesis, neurogenesis, neurodegeneration, hippocampus, Wnt signaling, antidepressants, mesenchymal stem cells, models of human hippocampal neurogenesis, interspecies differences, cell therapy

## Abstract

The hippocampus is one of the few privileged regions (neural stem cell niche) of the brain, where neural stem cells differentiate into new neurons throughout adulthood. However, dysregulation of hippocampal neurogenesis with aging, injury, depression and neurodegenerative disease leads to debilitating cognitive impacts. These debilitating symptoms deteriorate the quality of life in the afflicted individuals. Impaired hippocampal neurogenesis is especially difficult to rescue with increasing age and neurodegeneration. However, the potential to boost endogenous Wnt signaling by influencing pathway modulators such as receptors, agonists, and antagonists through drug and cell therapy-based interventions offers hope. Restoration and augmentation of hampered Wnt signaling to facilitate increased hippocampal neurogenesis would serve as an endogenous repair mechanism and contribute to hippocampal structural and functional plasticity. This review focuses on the possible interaction between neurogenesis and Wnt signaling under the control of antidepressants and mesenchymal stem cells (MSCs) to overcome debilitating symptoms caused by age, diseases, or environmental factors such as stress. It will also address some current limitations hindering the direct extrapolation of research from animal models to human application, and the technical challenges associated with the MSCs and their cellular products as potential therapeutic solutions.

## 1. Introduction

The hippocampus plays a critical role in the process of neurogenesis, since it is one of the unique regions in brain where new functional neurons are generated from neural stem cells (NSCs) throughout the life-span [1,2,3]. Hippocampal neurogenesis is associated with the dentate gyrus (DG) in the adult hippocampus, where new neurons are derived from progenitor cells that reside in the layer called the sub-granular zone (SGZ). In the adult hippocampus new granule cells are generated from this layer. During the neuronal differentiation cascade in the DG, the quiescent neural progenitors (QNPs) generate amplifying neural progenitors (ANPs) through approximately three asymmetric divisions. These ANPs exit the cell cycle and differentiate into neuroblasts and then into immature neurons (INs) and differentiated mature neurons after two rounds of symmetric division [4,5].

Distinct NSC populations respond differently and selectively to neurogenic stimuli and pathological conditions [6,7,8]. The first NSC population contains the glial fibrillary acidic protein (GFAP)-expressing radial glia-like cells which divide and generate new neurons under normal conditions or after the chemical removal of actively dividing cells [6]. The second NSC population displays active horizontal morphology in response to mitotic signals and is lost or exhausted with age [8]. A loss of active horizontal NSCs correlates with reduced neurogenesis in aged mice.

**Figure 1:**
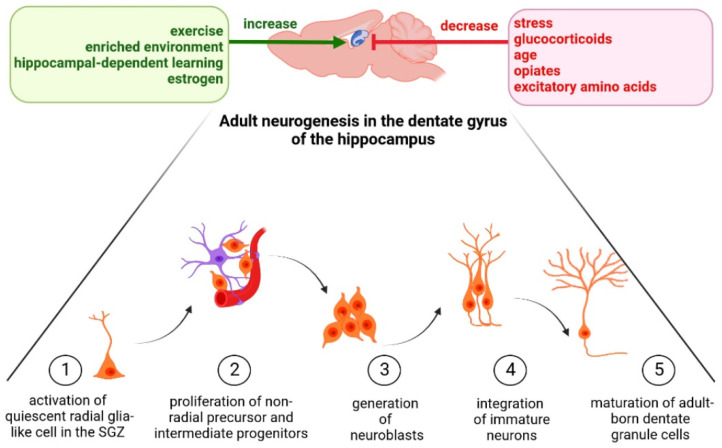
Changes in adult hippocampal neurogenesis by numerous factors (*The figure was made in Biorender*).

Adult hippocampal neurogenesis is dynamically regulated by numerous external factors and intracellular pathways under physiological and pathological conditions [9]. Several stimuli change the composition of the cellular and molecular neurogenic niche in which the adult neural stem cells exist, affecting their proliferation and differentiation [10,11] and defining the final hippocampal structure and function. Neurogenesis can be stimulated by exercise, an enriched environment, hippocampal-dependent learning and estrogen, while being decreased by stress, glucocorticoids, age, opiates and excitatory amino acids [11,12,13,14,15,16,17,18]. This has been presented in Figure 1.

There is consistent evidence of a dose-response relationship between stressful life events and depression [19], with the latter being associated frequently with small hippocampal volumes [20]. Since the hippocampus is a pivotal brain structure in the pathophysiology of stress-related diseases, there is potential for it to remodel upon exposure to stress. It was observed that both repeated restraint stress and repeated corticosterone treatment may result in atrophy of dendrites of hippocampal neurons [21,22]. Furthermore, suppression of the neurogenesis of DG granule neurons, which causes atrophy of dendrites in the CA3 region, was also observed after exposure to stress [23]. However, the effect of stress on hippocampal neurogenesis is an individualized process which is determined by individual exposure to stressful life events. Therefore, an inhibition of adult neurogenesis only leads to a measurable decrease in hippocampal volume and does not seem to be responsible for all hippocampal volume loss that occurs after exposure to stress [24]. Especially, given that stress can induce both structural and functional changes in a heterogeneous way at the cellular and whole organism levels. 

The proliferation of progenitor cells and subsequent developmental events such as dendritic growth, axon elongation, and synapse formation define the adaptive structural plasticity in the hippocampus [25]. Given this crucial definition, the abnormalities in hippocampal structure combined with the dysfunction of hippocampal neurogenesis observed in many mood disorders such as posttraumatic stress disorder (PTSD), major depressive disorder (MDD), borderline personality disorder (BPD) and bipolar disorder (BD) [26,27] could find an association with the impairment of proliferation in progenitor and neural stem cells and defects in the control of cell differentiation in the adult DG. 

Administration of antidepressants restores neurogenesis in many animal models of mood disorders. Moreover, their antidepressant effect can be associated with modulation of the Wnt signaling pathway during the stimulation of hippocampal neurogenesis. On the other hand, it is known that transplantation of mesenchymal stem cells (MSCs) also activates the Wnt/β-catenin signaling pathway in the hippocampus. Therefore, the present review focuses on the possible interaction between neurogenesis and Wnt signaling under the control of antidepressants and MSCs.

## 2. Strategies for Influencing Neurogenesis in the Adult Hippocampus Based on the Wnt Signaling Pathway Modulation

In elegant studies, the stereotactic injection of lentivirus vectors (LV–GFP or LV–dnWnt (LV–dnWnt–IRES–GFP)) into the adult DG of the hippocampus blocked the Wnt signaling and stopped adult hippocampal neurogenesis almost completely in vivo [28]. Similarly, blocking Wnt (Wingless-related integration site) signaling by the dnLEF-1 (doxycycline-induced expression of lymphoid enhancer factor 1 which can mediate the expression of Wnt signaling genes via recruitment of β-catenin to the promoter of the target genes) reduced neurogenesis in adult hippocampal stem/progenitor cells (AHPs) in vitro [28]. It confirms that Wnt signaling pathway plays a master role during the generation of newborn neurons in the adult dentate gyrus both in vivo and in vitro [29].

### 2.1. Regeneration of Hippocampal Neurons by the Modulation of Wnt Signaling Pathway 

The canonical Wnt/β-catenin signaling plays an important role in balancing the self- renewal of NSCs and neuronal differentiation in adult DG [30] which regulates the plasticity of the hippocampal network. Thus, the activation of the Wnt/β-catenin pathway improves neurogenesis in the hippocampus. Activation of the Wnt/β-catenin pathway prevents the degradation of β-catenin and allows the transcription of Wnt-specific target genes [31]. It is accompanied by activation of Dishevelled (Dvl) by the Frizzled (Fz) receptor and inhibition of the cytoplasmic β-catenin destruction complex composed of the scaffold protein Axin, adenomatous polyposis coli (APC), and glycogen synthase kinase-3β (GSK-3β). The binding of Wnt ligands to their Frizzled receptors and co-receptors LRP5 and 6 (5/6) stabilizes cytoplasmic β-catenin by suppressing Axin function [32]. It is noteworthy that membrane recruitment of Axin sometimes seems to be sufficient to activate Wnt/β-catenin signaling, even in the absence of Fz or Dvl [33].

Interestingly, the canonical Wnt signaling acting via β-catenin stabilization is a key regulator of the blood-brain barrier (BBB) phenotype during embryonic and postnatal development [34]. The BBB dysfunction and higher concentration of albumin in the cerebrospinal fluid (CSF) are hallmarks observed in dementia patients [35] and in patients with several other neurological disorders. Furthermore, it was observed that stimulation of the Wnt/β-catenin signaling pathway by human serum albumin, which can cross the damaged BBB, improves neurogenesis of the hippocampus and, in general, the neurologic function of brains subjected to cerebral ischemia-reperfusion injury [36,37]. Albumin can appear to have a supervisory role in regulating hippocampal neurogenesis since it coordinates multidirectional interactions between the immune, endocrine, and serotonergic systems [38]. However, more research in this domain is necessary to ascertain the causal relationship between the multifaceted interactions of albumin and its role in augmenting hippocampal neurogenesis and recovery in brains subjected to ischemia-reperfusion injury.

Both neural stem cells (NSCs) and neural progenitor cells (NPCs) secrete Wnt modulators that bind to Fzs, which are seven pass transmembrane receptors, and co-receptors to trigger the canonical Wnt/β-catenin-dependent or the non-canonical β-catenin-independent pathways [39]. An activation of the non-canonical Wnt pathways may induce either an increase in intracellular calcium concentration (Wnt/Ca^2+^ pathway), which induces the activation of calcium/calmodulin-dependent protein kinase II (CamKII) and protein kinase C (PKC), or activate the c-Jun-N-terminal kinase (JNK) cascade and Rho-associated protein kinase (ROCK) in the Wnt/planar cell polarity (PCP) pathway, the second non-canonical β-catenin-independent pathway [40,41,42].

Among the 10 Frizzled (Fz) receptors encoded in the mammalian genome [43], the Frizzled 1, 5, and 9 (Fz1, Fz5 and Fz9) receptors have the highest expression in the adult hippocampal DG.

The Fz1 receptor is highly expressed in NSCs, neural progenitors and immature neurons in the adult DG. This receptor regulates the specific stages of adult hippocampal neurogenesis, necessary for neuronal differentiation and positioning of newborn neurons into the granule cell layer. However, it does not regulate the stages of adult hippocampal neurogenesis necessary for the morphological development of adult-born granule neurons [44]. 

The Fz5 receptor is present at the synapses and is upregulated during synaptogenesis in the hippocampus [45]. It has been observed that Fz5 is involved in the establishment of neuronal polarity and in neuronal morphogenesis in part through a non-canonical Wnt-JNK- dependent pathway in culture of rat hippocampal neurons isolated at embryonic day 18 (E18) [46].

The Fz9 receptor plays a key role in maintaining a balance between programmed cell death in the developing DG and the number of dividing precursors in the same sub-region of hippocampus [47]. It is known that Fz9 is one of about 28 genes typically deleted in individuals with Williams syndrome and is associated with a microdeletion in the long arm of chromosome 7 [47]. Since both an increased level of apoptosis and increased doubling time were observed in Williams syndrome (WS) induced pluripotent stem cell (iPSC)-derived NPCs [48], modeling Williams syndrome with iPSCs confirms the important role of this receptor in apoptosis, the latter decides about the hippocampal plasticity. 

Wnt ligands are mainly modulators of synaptic plasticity and have pleiotropic Fz signaling activities. Additionally, the mammalian genome encodes 19 Wnt proteins [41], resulting in numerous possible regulatory combinations. However, in most cases, the upregulation of Wnt3 alone is sufficient to increase neurogenesis from adult hippocampal stem/progenitor cells (AHPs) both in vitro and in vivo [28].

Stimulation of Wnt3a provokes substantial recruitment of Dvl, Axin, glycogen synthase kinase-3β (GSK3β) and β-catenin to the plasma membrane [49]. Subsequently, the activation of Dvl, a masterful conductor of complex Wnt signals, results in both sequestration of GSK3β from the cytosol into multivesicular bodies (MVBs), separating the enzyme from its many cytosolic substrates such as β-catenin and stabilization of β-catenin [50,51]. Consequently, the stabilized β-catenin translocates into the nucleus to enhance the expression of many target genes by activating the transcription factors LEF1, TCF1, 3, and 4 [52].

The activation of Dvl and the subsequent inhibition of GSK3β (after stimulation by Wnt3a) induce the differentiation and formation of axons in cultured hippocampal neurons. In addition, a complex of critical mediators of neuronal polarization: PAR3, PAR6 and atypical protein kinase C (aPKC) is formed resulting in stabilization and activation of aPKC [53]. It is important to remark that Wnt5a, a non-canonical Wnt, is also pivotal for axon outgrowth specification in hippocampal neurons via interaction with aPKC [53]. Moreover, the Dvl siRNA-induced phenotype (silenced Dvl) can be rescued by overexpressing aPKC [54]. Thus, the overexpression of aPKC plays a critial role in the polarization of a new-born neuron into its axonal domain.

The Wnt ligand Wnt7a is essential in multiple steps of adult hippocampal neurogenesis and regulates these processes by activating downstream target genes of the canonical Wnt-β-catenin pathway that are involved in cell cycle control under proliferating conditions (e.g., *cyclin D1*) and those involved in neuronal differentiation upon differentiation (*Neurogenin2*) [55]. It was confirmed in Wnt7a knockout mice that the ligand plays a more critical role in hippocampal neuronal development than in the impaired dendritic arborization of dentate granule neurons [55]. Loss of Wnt7a expression reduces neural stem cell self-renewal, increases the rate of cell cycle exit in neural progenitors, as well as inhibits hippocampal DG neuron production and maturation [55]. Furthermore, the discovery of the role Wnt7a has in determining neurotransmitter release and synaptic plasticity upon administration to cultured hippocampal neurons [56,57] should pique interest in exploring the equivalence of this phenomenon in vivo in addition to its specific role in long-term potentiation. This could significantly improve cognitive parameters such as learning and memory, which are often adversely affected in neurodegenerative disease. Additionally, Wnt7a has been found to be one of the key Wnt molecules expressed at the peak of synapse formation in the hippocampus, indicating a prominent role in the process [58].

Interestingly, restoration of Wnt7a was able to reverse a lung cancer transformation through Fz9 mediated growth inhibition and promotion of cell differentiation via activation of the Wnt/JNK pathway [59]. In turn, in the intestinal niche, MSCs have been found to secrete Wnt molecules which are directly involved in canonical Wnt signaling [60]. This suggests that the microenvironmental context can drive the regulatory networks that control the adult hippocampal neurogenesis. Therefore, it could be feasible to consider the possibility of some of the directly transplanted MSCs in the sub-granular microenvironment to behave similarly and modulate Wnt signaling there to facilitate hippocampal neurogenesis. This, in turn, could either lead to supplementation in the event of deficient endogenous Wnt signaling with the supplemental Wnts doubling as agonists or prompt the restoration of Wnt signaling if the endogenous function is completely absent (which could be investigated in knockout models).

While Wnt7a has a limited role in impaired dendritic arborization of dentate granule neurons, Wnt7b, which is also expressed in the hippocampus, increases dendritic arborization in hippocampal neurons and accelerates dendritic development by increasing neurite length and branching [61] by Wnt/β-catenin signaling [62]. Interestingly, the non-canonical Wnt7b-Dvl signaling pathway also regulates dendritic development through the activation of Rac and JNK [61].

Overall, the above studies confirm that both canonical and non-canonical Wnt signaling pathway can be engaged in the stimulation of hippocampal neurogenesis.

**Figure 2:**
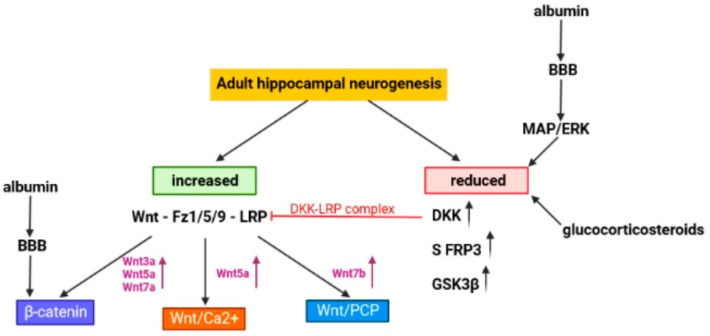
Strategies for influencing neurogenesis in the adult hippocampus based on the modulation of the Wnt signaling pathway (*The figure was made in Biorender*).

This has been presented in Figure 2.

### 2.2. Decreasing Neurogenesis in the Adult Hippocampus by the Modulation of Wnt Signaling Pathway

The activation of the Wnt/β-catenin pathway improves neurogenesis in the brain and this neuroprotective effect can be negatively regulated by antagonists of this pathway, e.g., Dickkopf-1 (DKK1), secreted frizzled related protein 3 (sFRP3) [63,64]. A decrease in canonical Wnt signaling activity in the DG reduces adult hippocampal neurogenesis and this correlation is observed in aging [29].

DKK1 is one of the best studied and characterized secreted inhibitors of the Wnt signaling pathway. This antagonist is a high affinity ligand for the Wnt co-receptor low density lipoprotein receptor–related protein 6 (LRP6). It inhibits Wnt signaling by binding to LRP6 and preventing Fz-LRP6 complex formation [65]. Both LRP6 function as a Wnt co-receptor for Fz proteins and DKK-1 inhibition of Wnt signaling by preventing Fz-LRP6 complex formation seem to be specific for Wnt/Fz signalling toward the β-catenin pathway [65]. Notably, DKK1 loss in NPCs increases neurogenesis in the SGZ of young and old animals and increases Wnt activity in NPCs, leading to enhanced self-renewal and/or survival of the more quiescent neural progenitors and increased numbers of amplifying neuronal progenitors (ANPs) and doublecortin (DCX)-positive neuroblasts (Nbs) [66].

Interestingly, DKK1 expression, which increases with age [66], can be regulated in a manner independent of Wnt signaling [52]. Moreover, given that DKK1 is overexpressed in Alzheimer’s brain [67] while the activation of Wnt signaling rescues neurodegeneration [68], the inhibition of Wnt signaling by DKK1 might be a critical factor in neuronal dysfunction associated with dementia. The latter is observed in Alzheimer’s disease (AD) pathology [69]. It is known that there is a strong association between DKK1, albumin and AD since a significantly decreased level of albumin is associated with over-expression of DKK in Alzheimer’ disease [69]. Given that albumin is a secret peripheral factor that controls the level of brain and peripheral serotonin [38] and the activation of mitogen-activated protein kinase (MAPK) is observed after induced dysfunction of the serotonergic system [70,71], the serotonergic system can control neurodegeneration by activating the MAP kinase pathway and through the Wnt/β-catenin independent pathway. In line with this, the MAP kinase pathway is engaged in the regulation of DKK1 expression but not the Wnt/β-catenin pathway [52]. Overall, these studies suggest that albumin may also supervise the control of neurodegeneration in hippocampal neurons. This hypothesis is also supported by an observation that activation of GSK3*β* via phosphorylation at Tyr279 engages the MAP kinase pathway after stimulation with serum albumin [72]. It is worth noting that the hyperactivation of GSK3*β*, a key component of the β-catenin destruction complex, is associated with dysregulated serotonergic activity [73].

Interestingly, a single injection of lentivirus-expressing GSK-3β shRNA in the hippocampal DG of chronically stressed mice has antidepressant-like effects elicited by gene silencing [74]. In turn, transgenic mice over-expressing GSK-3β recapitulate some of the symptoms (such as hyperactivity) observed in the manic phase of bipolar disorder patients, ADHD, and schizophrenia [75]. However, they have also an elevated level of brain-derived neurotrophic factor (BDNF) in the hippocampus [75]. This suggests that a mechanism is triggered to resolve symptoms of hyperactivity and mania associated with BDNF. It is known, that BDNF administration to the DG leads to increased neurogenesis of granule cells [76]. Moreover, cAMP response element-binding protein (CREB) activation results in elevated levels of BDNF protein, with the latter assumed to produce at least some of the antidepressant drug activity in animal models [77]. Furthermore, it is known that antidepressants are used to treat depressive symptoms in patients with depression which can be a consequence of strong stress. Although the pathogenesis of depression is not fully known, it is noteworthy that research into disorders associated with depression will help us to find a way to boost neurogenesis in the adult hippocampus by indicating a strategy to combat dementia.

## 3. Stress as an Environmental Factor Regulating Hippocampal Neurogenesis

Stressful life situations and experiences can lead to depression e.g., postpartum depression [78]. Studies in the hippocampus have shown that stress can affect adult brain plasticity through neurogenesis [79]. Exposure to stressful experiences as well as chronic stress results in a significant reduction in the number of proliferating cells in the DG [79] and decreases neurogenesis in the adult hippocampus, which is an important brain structure in the pathophysiology of stress-related diseases because it mediates both cognitive deficits and hypercortisolemia found in these disorders [80].

The Wnt signaling pathway is modified as a consequence of stress. Furthermore, an inhibition of Wnt signaling during the chronic stress plays a key role in extinguishing adult hippocampal neurogenesis. Mice exposed to mild restraint stress show a reduction in hippocampal volume associated with neuronal loss and dendritic atrophy in the CA1 region, and a reduced neurogenesis in the DG [81]. Chronic restraint stress exposure selectively decreases Wnt2 and Wnt3 expression in the hippocampal DG [82] and leads to a reduction in the expression of hippocampal β-catenin, an intracellular protein positively regulated by the canonical Wnt signalling pathway [81]. Simultaneously, an increased level of DKK1 is observed in the hippocampus. DKK1 antagonises Wnt signalling by disassembling synapses through the activation of the GSK-3 and Rock pathways. The disassembly and consequent loss of the synapses leads to synaptic plasticity deficits and impairments in long-term memory [83]. The stimulation of glucocorticoid receptors mediates this effect in response to chronic stress [82].

It is known that the hippocampus contains a high density of glucocorticoid receptors [84] which can be exposed to elevated levels of glucocorticoids during chronic stress. An increase in the amount of the neuron-specific phosphoprotein synapsin I in the hippocampus is observed after the administration of glucocorticoids [85]. Moreover, increases and decreases of synapsin I in the DG/CA3 and CA1, respectively, have also been observed in the hippocampus in a rat model of depression induced by chronic unpredictable mild stress [86]. Furthermore, administration of the glucocorticoid receptor antagonist, mifepristone (RU-486) decreases the level of synapsin I in the DG/CA3 while increases in the CA1 subfield in this model [86]. Owing to this association, stress and glucocorticoids decrease neurogenesis through dendritic shrinkage and loss of spines in the hippocampus. They also suppress hippocampal BDNF [87].

Surprisingly, the anti-inflammatory actions of glucocorticoid receptors seem to be important in the stimulation of neurogenesis. Especially considering that inflammatory changes are consistently observed after exposure to stress and some of them, such as an increase in the levels of IL-1β and decrease the cell proliferation in the hippocampus [88]. Exposure to glucocorticoids releases glucocorticoid receptors from the heat shock complex with the specific dissociation of GSK3α [89]. GSK3α selectively inhibits IL-1-induced activation of mTOR signaling [72]. Moreover, GSK3α can inhibit the mTOR pathway independently of β-catenin [90], a key mediator of the canonical Wnt signaling pathway. Furthermore, Wnt-mediated GSK3α regulation can also modulate the regulation of SMAD1 independently of β-catenin [90].

In summary, the selective glucocorticoid receptor stimulation by the elevated glucocorticoid levels which are observed during stress seems to play a bi-phasic role in the regulation of hippocampal neurogenesis by engaging the Wnt signaling pathway.

## 4. The Influence of Wnt Signaling Pathway on the Interaction between Hippocampal Neurogenesis and Learning

Neurogenesis and learning are linked by a feedback mechanism. Learning influences neurogenesis in a complex manner and neurogenesis determines learning performance and memory [91] indicating a complex, yet substantial interdependence. The rate of proliferation, differentiation, and survival of newborn neurons in the hippocampus can be modulated by hippocampus-dependent learning. It is known that the hippocampus plays an important role in the processing of spatial information [92]. It has also been observed that prenatal stress can block the increase of learning-induced neurogenesis in the DG and impairs the hippocampus-related spatial tasks in adult rats [93]. Additionally, administration of fluoxetine during adolescence impairs spatial memory in adult male, but not female, C57BL/6 mice [94]. There is a direct association between hippocampus-dependent learning of spatial tasks and neurons generated in the adult hippocampal formation. This has been established in rats which have shown a doubling of adult-generated neurons in the DG in response to training on associative learning tasks requiring the hippocampus [95]. Loss of DKK1, an inhibitor of the canonical Wnt pathway in NPCs residing in the SGZ of the DG has a positive effect on hippocampus-dependent spatial working memory and increases both hippocampal neurogenesis as well as Wnt activity in the hippocampus [66]. Specifically, an increase in Wnt5a and Wnt7 levels restricted to hippocampal granule cells level after spatial learning is observed whereas the Wnt3 level remains unchanged [96]. In contrast, the DG-specific knockdown of adult neurogenesis by inhibiting Wnt signaling is sufficient to impair spatial memory in adult rats [97]. This is interesting since Wnt3 triggers the expression of insulin [98] which can be important in insulin/IGF-1 signaling for spatial learning and memory in the hippocampus. The inactivation of both insulin receptors (IRs) and IGF-1 receptors (IR/IGF1R) in the hippocampus, on the contrary, leads to impaired spatial memory [99]. Moreover, conditional ablation of the type 1 insulin-like growth factor (IGF-1) receptor gene (*Igf1r*) results in an almost complete loss of the DG in mice [100]. While in contrast, peripherally administered IGF-1 increases neurogenesis in the adult DG [101]. Furthermore, the concentration of IGF-1, which controls brain plasticity [102], is also associated with the regulation of albumin concentration [103]. Finally, it is worth mentioning that the clinical intranasal administration of IGF-1 appears to be a plausible and promising treatment option for depression [104]. Owing to these associations, we can hypothesize that, IGF-1 can provoke an interaction with albumin to enhance neurogenesis in the hippocampal DG.

To conclude, these studies confirm that adult hippocampal neurogenesis can be stimulated without direct interaction with the hippocampus.

## 5. Boosting Hippocampal Neurogenesis by Mood Stabilizers and Antidepressant Treatment

Drugs which are mood stabilizers or antidepressants increase an adult hippocampal neurogenesis [105,106,107,108]. Interestingly, the antidepressant-induced upregulation of cell proliferation is specific to the hippocampal DG because chronic electroconvulsive seizures (ECS) or fluoxetine treatment do not influence the number of BrdU-labeled cells in another brain region known to contain progenitor cells in adulthood, the sub-ventricular zone of the lateral ventricle [105]. Moreover, antidepressant-induced neurogenesis seems to be antidepressant-specific based on the modification of neurogenesis mechanism. For example, fluoxetine-induced neurogenesis has been observed to differ from imipramine-induced neurogenesis [109]. Chronic treatment with fluoxetine, a selective serotonin reuptake inhibitor (SSRI) increased BrdU (a marker of DNA synthesis)-positive cells only in WT (wild type) mice but had no effect in KO (5-HT1A receptor knockout) mice. In turn, chronic treatment with imipramine (a classic, tricyclic antidepressant which is a mixed serotonin/noradrenaline reuptake inhibitor) induced a significant increase in BrdU-labeled cells in both WT and KO mice.

On the other hand, fluoxetine administration decreases neuronal apoptosis [110,111] and promotes neurogenesis in the hippocampal DG by up-regulating Wnt3a and CREB, with the latter being positively associated with enhanced mitosis rates in progenitor cells [112]. Since CREB activation is required for BDNF expression in hippocampal neurons [113], it is not surprising that fluoxetine can increase the BDNF expression [110]. The selective loss of BDNF in the DG results in an attenuation of antidepressant efficacy [114]. Imipramine can also induce BDNF expression through CREB activation via PKA and/or ERK pathways [115]. Moreover, increased expression of CREB mRNA and CREB functional protein in response to chronic treatments with fluoxetine or imipramine is observed in the same sub-regions of the hippocampus where the regulation of BDNF and trkB take place (i.e., DG granule cell layer and the CA3 and CA1 pyramidal cell layers) [77].

Overall, these studies suggest that the noradrenergic system can compensate for the effect of the serotonergic system during the stimulation of hippocampal neuron regeneration and concentrate our attention on an action on second messenger systems associated with these monoaminergic systems.

Unimpaired hippocampal neurogenesis is often required for the effective action of antidepressants. This is clearly evident in the observation of behavior which indicates antidepressant-like effects after electroconvulsive stimulation (ECS) [116]. The physiological modulation of neurogenesis by ECS is associated with sFRP3 downregulation [117]. Chronic treatments with imipramine or fluoxetine also significantly suppressed a Wnt signaling inhibitor, sFRP3 expression in the hippocampus [118]. Additionally, fluoxetine-treated adult sFRP3 KO or heterozygous (HET) mice showed significantly reduced levels of immobility compared to WT littermates in tail suspension and forced swimming tests, which are two models for assessing antidepressant activity [118,119,120].

Interestingly, the effect of fluoxetine on adult hippocampal neurogenesis seems to be inconsistent in vivo given the large difference depending on the route of drug administration. Intraperitoneal or osmotic pump facilitated administration allowed the confirmed observation of change in hippocampal neurogenesis through an increase in the number of BrdU and Ki67 positive cells in the DG [105,121,122]. However, administration via oral gavage did not always demonstrate this effect [123,124,125]. It needs to be highlighted that fluoxetine acts on the basis of a powerful neurogenic effect on DG progenitor cells, but is ineffective on stem cells [126]. Moreover, fluoxetine inhibits GSK3β in the hippocampus [110]. Additionally, GSK3*β* in cooperation with CREB mediates suppression of cyclin D2 expression [127], which is important for adult neurogenesis [128]. It is equally noteworthy that the over-expression of GSK3β in the hippocampal DG results in depressant-like effects which can be reversed by chronic fluoxetine administration [111].

Analogously, chronic administration of lithium, a mood stabilizer, increases neurogenesis in the DG of adult rodents [108]. Lithium also increases rates of hippocampal progenitor cell differentiation [129] and inhibits GSK3β directly [130]. Moreover, increased recovery in the levels of β-catenin and Dvl-3 along with increased GSK3β inhibition has been observed after lithium treatment in APP-PST1 (a mouse model of Alzheimer’s disease) mice [131].

All these associations confirm that the effect of fluoxetine on adult hippocampal neurogenesis is evident. Additionally, BrdU and Ki67-positive cell were also observed in significantly increased numbers in the hippocampal DG of mice modeling Angelman syndrome with an imprinted expression of *Ube3a*, after chronic fluoxetine treatment [132]. Deficient *Ube3a* expression decreases the number of NeuN/BrdU-positive cells [133].

Interestingly, *Ube3a* expression deficiency may result in impaired of GR signaling [133]. It is known that the rate of adult hippocampal neurogenesis in the DG is dependent upon circulating glucocorticoid levels during adulthood [13] as they tend to suppress the number of newly generated cells [134]. It is known that increased glucocorticoid secretion is a key feature of the stress response [135]. Besides, glucocorticoids regulate 5-HT1A receptor mRNA in the hippocampus [136] and restraint stress significantly decreases the binding of serotonin to 5-HT1A receptors in the DG [137]. Furthermore a variation in the level of glucocorticoids following a diurnal pattern is necessary for fluoxetine to stimulate neurogenesis in the adult DG [122]. This confirms that the effect of fluoxetine on hippocampal neurogenesis depends on the level of glucocorticoids.

It is noteworthy that fluoxetine competes with glucocorticoids to bind to human serum albumin and induces conformational changes in albumin structure [138]. Surprisingly, imipramine also competes directly with tryptophan to bind to human serum albumin [139] and changes its structural conformation [140]. Also, the altered conformation of albumin may be responsible for decreased binding of tryptophan in the plasma, which ultimately results in changes in serotonin levels despite the same level of albumin [38]. Furthermore, alterations in the conformation of serum albumin appear to be a mechanism to control tryptophan levels in the brain [38], consequently influencing serotonin levels (5-hydroxytryptamine, 5-HT).

Albumin is a long-term strategically important physiological factor that plays a key role in the stimulation and control of serotonin synthesis and its levels in the brain and its periphery [38]. It could imply that both inhibition of 5-hydroxytryptamine (5-HT) synthesis (by intraperitoneal administration of para-chlorophenylalanine, PCPA) and selective lesions of 5-hydroxytryptamine neurons (by intracerebral administration of 5,7-dihydroxytryptamine, 5,7-DHT) are associated with decreases in the number of newly generated cells in the DG [141] as a consequence of albumin and its influence on serotonin synthesis. It was observed that therapy based on a high-concentration of human serum albumin is beneficial in reducing both the neurobehavioral and histopathological consequences of experimental traumatic brain injury (TBI) [142]. TBI has been proven to enhance neural stem cell (NSC) proliferation in the hippocampal DG [143]. Furthermore, a high-dose of serum albumin therapy administered within five minutes after global ischemia significantly improves neurological scores and reduces histological damage [144].

Interestingly, a stroke which occurs as a consequence of focal ischemia induces the formation of new neurons in the adult rodent brain from neural stem/progenitor cells (NSPCs) located in two regions: the subventricular zone (SVZ) lining the lateral ventricle, and the SGZ in the DG [145]. However, stroke-induced increase of neurogenesis in the DG is attenuated in an aged brain [146].

Considering these results together, we can conclude that successful stimulation of adult hippocampal neurogenesis depends on the action of second messenger systems associated with the 5HT1A-dependent enhancement of serotonergic neurotransmission under the control of glucocorticoid receptors. Moreover, augmented dosage of antidepressant medication also has a strong association with the stimulation of hippocampal neurogenesis.

## 6. Influence of Chronic Antidepressant Administration on Adult Hippocampal Neurogenesis via the Wnt Signalling Pathway

The stimulation of the hippocampal neurogenesis associated with an antidepressant effect via Wnt signalling pathway may be achieved in different ways. One binds to Wnt ligands and can promote long-term antidepressant effect [82], while the other aims to increase Axin expression [147].

Chronic administration of SSRIs (citalopram or fluoxetine), a selective norepinephrine (NE) reuptake inhibitor (SNRI, atomoxetine) or a dual 5-HT/NE reuptake inhibitor (venlafaxine) influences the hippocampal expression of multiple components of Wnt signaling pathway including Wnt2, Wnt7b, Fz9, Frzb, Dvl1, TCF15, TCFL1, LEFT1, NeuroD1, β-catenin, and Akt1 [148]. A common target of these different classes of antidepressants is Wnt2 and its increased expression is observed in the hippocampal DG only after chronic administration of fluoxetine or venlafaxine [148]. Moreover, it was observed that repeated administration of ECS increased the levels of β-catenin immunoreactivity in the SGZ of the hippocampus and significantly increased levels of Wnt2, one of the ligands that activate β-catenin [149]. The increased Wnt2 participates in regulating the patterning and outgrowth of the hippocampus through nuclear mediators of Wnt signaling such as the TCF/LEF1 proteins [150]. Additionally, the increased expression of Wnt2 is sufficient to produce the antidepressant-like behavioral effect in different models of depression [148]. However, the question of Wnt2’s ability to regulate hippocampal neurogenesis lingers?

Wnt2 is a physiological ligand of the Fz9 receptor [151]. It has been observed that co-expression of selectively expressed in the hippocampus Fz9 with each of the Wnt proteins resulted in a 10-fold increase of TCF activity only in the case Wnt2 [151]. All other Wnt proteins, including Wnt-1, -3, -3a, -4, -5a, -5b, -7a, and -7b tested with Fz9 showed no significant increase of TCF activity [151]. Since Fz9 can be specifically activated by Wnt2 [151] and Wnt3a promotes NPC proliferation in the DG [152], as well as an association between their expression profiles can be established [82], then we can deduce that Wnt2 has the ability to regulate hippocampal neurogenesis. Moreover, the transcription of *Wnt2* is CREB-dependent [153]. In addition, fluoxetine can increase the transcriptional activity of CREB [82] which has an important role in pathophysiology and treatment of depression. These considerations lead to the conclusion that fluoxetine can activate the Wnt/β-catenin signaling by stimulating the interaction between Wnt2 and Wnt3. Consequently, the converse, which will imply the loss of the antidepressant effect of fluoxetine upon ablation of Wnt2 or Wnt3 also holds true [82]. Furthermore, a 14 day administration course of fluoxetine promotes neurogenesis in the hippocampal DG by up-regulating *Wnt3a* and *CREB* [112]. In addition, an intra-cerebroventricular (ICV) infusion of the Trk antagonist K252a leads to upregulation of *Wnt3a* alone after 14 days of fluoxetine treatment [112].

**Figure 3:**
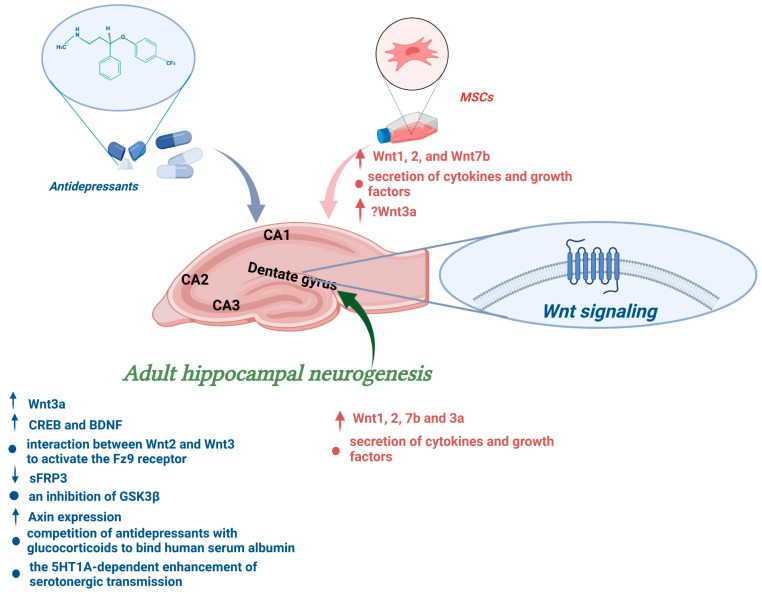
Influence of antidepressants and mesenchymal stem cells on adult hippocampal neurogenesis via the Wnt signaling pathway (*The figure was made in Biorender*).

Considering all these results together, we can conclude that antidepressants provoke an interaction between different components of the Wnt signaling pathway to stimulate neurogenesis in the adult hippocampus. This has been presented in Figure 3.

## 7. Mesenchymal Stem Cells Can Open the Plasticity Window in Personalized Therapy

The therapeutic potential of MSCs is mainly dependent on their neuro-protective and immunomodulatory properties due to paracrine acting molecules. However, the efficacy of MSCs depends on secreted factors released by MSCs themselves or by other cells of the niche that were stimulated by MSCs. In these two ways, MSCs express a variety of neuro-regulatory proteins such as BDNF, GDNF, VEGF, IGF-1, FGF-2 or β-NGF, [154,155,156,157] affecting the damage niche that the surrounding target cells. It is noteworthy that the efficacy of mesenchymal stem cell transplantation depends on different factors such as the optimal route, dosage, and timing of stem cell treatment as well as the type of stem cell being administered [158]. All these factors can influence the anti-inflammatory and neurorestorative properties of MSC.

The examples listed below show different efficacy of MSC treatment depending upon the administration route. The intravenous administration of MSCs post-injury significantly improved blood–brain barrier (BBB) function in vivo in a rodent model of traumatic brain injury (TBI), despite the lack of large numbers of MSCs in the brain [159]. Intracerebroventricular (ICV) injection of MSCs overexpressing FGF21 (MSC-FGF21) in a mouse model of TBI partially restored TBI-induced deficits in neurogenesis and maturation of immature hippocampal neurons [160]. Intraventricularly delivered MSCs using an external ventricular drain (EVD) significantly reduced expression of IL-1α, IL-6, and IFN-γ and alleviated brain damage in an intracerebral hemorrhage ICH rat model [161]. The intrahippocampal transplantation of allogeneic bone marrow-derived mesenchymal stem cells into the hippocampus of rats had long-term effects on hippocampal plasticity. Cell proliferation and neurogenesis in the DG were increased for more than 50 days after MSC transplantation, despite short-term graft survival in the brain [162]. Transplantation of human MSCs into ipsilateral brain parenchyma of rats with middle cerebral artery occlusion (MCAO) increased the expression of BDNF, NT-3, and VEGF in the ischemic rat brain and improved endogenous neurogenesis after cerebral ischemia in rats [163] While intravenously infused stem cells migrated to the spleen in rats with MCAO stroke [164] and indicated preferential migration to the spleen over the brain in adult rats with chronic stroke as evidenced by in vivo and ex vivo imaging analyses [165].

Both human and mouse MSCs are capable of differentiation into cells resembling immature neurons and expressing several neural proteins after exposure to specific experimental culture conditions [166]. Furthermore, the administration of cell-free exosomes generated by hMSCs cultured under 2D and 3D conditions increased the number of newborn mature neurons in the DG and improved spatial learning [167].

It is worth mentioning that the culture conditions of stem cells substantially influence their therapeutic value. Preconditioning in physiological oxygen levels (5% O_2_) and 3D culture increase their survival and neuroprotective properties by stimulating the secretion of anti-inflammatory cytokines and neuroprotective factors [168,169]. Similarly, preconditioning in 0.5% O_2_ enhanced the capacity of the secretome obtained from cultured human MSCs to release HGF and VEGF [170]. Moreover, VEGF increased neurogenesis [170]. The following ex vivo studies with indirect co-culture of organotypic hippocampal slices and cell-hydrogel bio-constructs revealed a strong neuroprotective effect of WJ-MSCs against neuronal death in the CA1 region of the rat hippocampus [168]. Furthermore, the secretome obtained from MSCs cultured in 0.5% O_2_ or 20% O_2_ blocked brain damage and functional impairtment in an adult rat model of TBI [170]. Interestingly, the viability of MSCs drastically decreases after the fourth day of culture [171]. Long-term neurosphere cultures (of three weeks and beyond) revealed reduced proliferation and clonogenicity. Consequently, this increases their proclivity towards faster senescence [171]. Thus, we can direct MSCs to acquire the required therapeutic properties by applying appropriate culture conditions.

The promising in vitro results do not always translate into in vivo studies. The level of BrdU-positive cells in the hippocampus of animals treated with adipose-derived MSC spheroids 3D (sMSC) as compared to the adherent MSC (aMSC)-group was significantly lower [172]. However, sMSCs showed more promising results in vitro [172]. Moreover, the absolute numbers of newly generated cells in the dentate gyrus were significantly lower in the sMSC treated animals compared to the 6-hydroxydopamine (6-OHDA) treated group. On the other hand, restored dopamine (DA) function was observed in the 6-OHDA-lesioned animals after transplantation of adherent MSC (aMSC). These animals showed reduced rotational behavior [172]. Interestingly, transplantation of human MSCs into the hippocampal DG of immunodeficient mice stimulates proliferation, migration, and differentiation of endogenous NSCs that expressed the stem cell marker Sox2. It also enhances the expression of VEGF, CNTF, NT3, NT4, NGF by endogenous cells [173]. It is known that VEGF acts at an early (proliferative) phase of neurogenesis [174]. Moreover, VEGF also stimulates neurogenesis both in mouse brain cultures in vitro and in neuroproliferative regions SGZ of the nonischemic mouse brain in vivo [175].

Apart from the many pro- and anti-inflammatory cytokines and growth factors secreted by MSCs such as IL1α, IL1β, IL2-15, TNFα, TNFβ, TGFβ, BDNF, VEGF, IGF-1, PGE2, GDNF, IGF-1, FGF-2 or β-NGF [154,155,156,176,177], these cells also produce a number of canonical Wnts such as Wnt1, 2, and 7b or stimulate the synthesis of Wnt3a by other cells after contact with MSCs [178,179,180,181,182], which in turn modulate the Wnt signaling pathway. In line with this, it has been observed that MSC administration significantly augments hippocampal neurogenesis and increases the differentiation of NPCs into mature neurons in AD models by augmenting the Wnt signaling pathway [183]. Moreover, intravenously administered MSCs resulted in an increase in both circulating and hippocampal Wnt3a with simultaneous activation of the Wnt/β-catenin signaling pathway in the hippocampus in a mice model of TBI [182]. Additionally, recombinant Wnt3a treatment largely but not completely recapitulated the effects of MSCs and improved performance in behavioral tasks that require hippocampal neurogenesis [182]. Furthermore, administration of Zeb2/Axin2-enriched exosomes derived from MSCs transfected with a Zeb2/Axin2 overexpression plasmid to MCAO rats exhibited a significant increase in the total number of BrdU- and NeuN-positive cells in the hippocampus [184].

Transplantation of human MSCs into the DG of the hippocampus of mice stimulates the proliferation, migration and neural differentiation of endogenous neural stem cells [173]. Notably, human MSCs implanted into the DG disappear over 5 to 7 days in both immunodeficient and immunocompetent mice [173]. Thus, MSC accumulation over a short time after transplantation increases their safety. On the other hand, it seems that the short accumulation time of MSCs may also influence their effectiveness. However, it has been shown that MSCs promote the endogenic neurogenesis, stabilize the neural network in the hippocampus and improve spatial learning and memory ability in aging mice [185]. Moreover, the administration of human MSCs up-regulates plasticity-related genes, reverses spine loss and promotes synaptic plasticity via ERK-CREB pathway in the aged hippocampus [185]. Thus, successful stimulation of adult hippocampal neurogenesis by MSCs depends on an action on second messenger systems stimulated by MSC secreted factors.

Interestingly, IGF-1 or BDNF administration initiates a long-lasting cascade of neurochemical effects involving increased serotonin levels resulting in enduring antidepressant-like behavioral effects [186,187]. It suggests that MSCs have antidepressant properties associated with their secreted growth factors. In conjunction, it has been observed that an ICV injection of MSCs leads to an increase in hippocampal neurogenesis and production of antidepressant effects [188]. However, intra-hippocampal transplantation of MSCs promotes hippocampal neurogenesis alone without the antidepressant effects [162]. It suggests the antidepressant-like effect of MSCs has an indirect relationship with MSC-secreted factors. In line with this hypothesis, it has been observed that the soluble factors (e.g., PGE2, TGF-β) secreted by MSCs can suppress the local inflammation associated with the immune response of mast cells. In fact, mast cells (MCs) are known to synthesize serotonin in humans [189] and are the first source of proinflammatory signals released from non-neuronal cells during brain injury [190]. It suggests that stimulation of mast cells (MCs) by MSC-secreted factors plays a key role in achieving the antidepressant-like effect observed after MSC transplantation. However, it must be underlined here that stimulation of mast cells is a double edged sword for neurogenesis. Mast cells contain their very specific proteases which play both protective and detrimental roles in inflammation [191]. Notably, the inflammatory environment in the brain enhances the proliferation of newborn hippocampal neural precursor cells (NPCs) but also reduces the capacity of these cells to generate mature neurons and gives prominence to gliogenesis over the long-term [192]. Consequently, anti-inflammatory therapy restores adult hippocampal neurogenesis after endotoxin-induced inflammation and augments neurogenesis after cranial irradiation [14].

The beneficial therapeutic effect of MSCs can be enhanced by the BBB leak observed in many neurological disorders which allows the entry of cytokines and growth factors secreted by MSCs into the CNS and affects hippocampal neurogenesis. In the rat model of AD which serves as a good example, MSCs migrate across the BBB into the hippocampus after an intravenous injection and improve synaptic function and neurogenesis via the up-regulation of synaptophysin and GAP43 protein expression levels, respectively [193]. Furthermore, MSCs modulate endothelial cell (EC) permeability both in vitro and in vivo in a rodent model of traumatic brain injury (TBI) [159]. Co-culture of MSCs with ECs decrease paracellular permeability by a secreted factor released as a result of MSC–EC interaction in vitro. This effect requires VE-cadherin/β-catenin interaction [159]. Further study confirmed that MSCs promote vascular stability after TBI through increased production of the tissue inhibitor of matrix metaloproteinase-3 (TIMP3), a protein that inhibits VEGF-A-induced compromise of the endothelial barrier [194].

Overall, these studies confirm that MSCs have the power to open the plasticity window of the hippocampal network and safely boost adult neurogenesis. This has been presented in Figure 3.

## 8. Possible In Vitro and In Vivo Models of Human Hippocampal Neurogenesis—Implications of Interspecies Differences

Adult hippocampal neurogenesis is a tightly orchestrated process which shows appreciable interspecies differences [195], implying that there will be some limitations while directly extrapolating animal results to humans. These species differences are visible both in the occurrence of adult hippocampal neurogenesis (e.g., absent in the hippocampus of bats) and in the effectiveness of brain repair after lesions or disease [195].

Sometimes detection of similar functional connectivity between brain structures in an animal model and in humans favors the extrapolation of results between species, despite the well-known obstacles in interspecies comparisons. Such a situation was observed after lesion of the dorsal hippocampus, directly implicated in spatial and episodic memory in rats [196], which revealed the mechanism of compensation for hippocampal damage in the medial prefrontal cortex (mPFC), the brain region that is not directly related to the site of injury [197]. Similar functional compensation between both brain structures is observed in incipient Alzheimer’s disease [198]. Thus, although the anatomical disparities of the rat brain structures compared to the human homolog are apparent, we are, nevertheless, able to observe similar functional connectivity between these two structures in both species.

On the other hand, human-based research conducted at the same time, can generate opposite conclusions. The article by Kempermann and colleagues aptly summarizes the opposite results from the same species obtained by two different laboratories at the same time [199]. Briefly, Sorrells and colleagues announced that the presence of young neurons in the human dentate gyrus declines with age to undetectable levels in adults [200]. Moreover, they observed a sharp drop in the number of proliferating progenitors and young neurons in the dentate gyrus during the first year of life. The opposite conclusion results from a simultaneous study conducted by another research group. Boldrini with colleagues observed that proliferating progenitor and immature neuron pools are stable with aging as well as the volume of the hippocampal dentate gyrus [201]. It is worth adding that Boldrini and colleagues assessed adult neurogenesis in human dentate gyrus between 14 and 79 years of age, implying they could not observe any human hippocampal neurogenesis drops during the first year of life.

Interestingly, data from the literature supports both opposing viewpoints. On the one hand, a study by Eriksson and colleagues with BrdU incorporation, which labels DNA during the S phase, detected the newborn neurons generated from a population of continuously dividing progenitor cells resided in the SGZ of the dentate gyrus in the adult human brain (average age of 64.4 ± 2.9 years) [2]. While on the other, radioactive carbon dating on sorted NeuN+ nuclei confirmed a restricted population of adult-born neurons in the adult human hippocampus and suggested that neurogenesis in the human SGZ continues throughout life [202]. Studies in the human SGZ using DCX and PCNA demonstrated an exponential decrease in neurogenesis in the SGZ in adults up to 100 years of age [203]. Another group showed that the density of Ki67+, DCX+ and Ki67+/DCX+ cells in the SGZ is high prior to one year of age, but then decreases markedly with age [204]. The conclusion of the above study was supported by progressive and significant decreases in mRNA levels of the proliferation marker Ki67 and the immature neuronal marker doublecortin (DCX) in the healthy human hippocampus over the lifespan [205]. In turn, analysis performed by Seki with colleagues using Ki67, HuB, and DCX antibodies showed that the number of proliferative neuronal progenitor cells was very low in the human DG [206]. In contrast, other studies demonstrated the detectable existence of hippocampal neurogenesis in many aged human brains throughout the lifetime using Nestin+Sox2+, PCNA+ and/or DCX, and NeuN markers [207,208] with only a mild decrease in the number of DCX1 immature dentate granule cells with age [207]. Notably, the extent of neurogenesis greatly varied between individuals [208]. The full list of studies confirming the occurrence of adult human hippocampal neurogenesis has been collected by Moreno-Jimenez and colleagues in their review paper [209].

Animal studies based on the stable incorporation of S-phase marker bromodeoxyuridine (BrdU) into the DNA of a dividing precursor cell and the later immunohistochemical visualization of BrdU in a neuron confirmed that adult neurogenesis is present in old Fischer 344 rats [210]. The study in 5- and 26-month-old Sprague-Dawley rats showed that the capacity for granule cell progenitor proliferation in the dentate gyrus of aged rats is the same as that in young rats and that the inhibition of neurogenesis in aged animals is the result of high corticosteroid levels [211]. In another study, thirty-eight-day-old and 12-month-old Sprague–Dawley rats experienced a nearly 94% reduction in neurogenesis relative to juveniles, due almost entirely to a 92% drop in cell production during the following two-month period [212]. In turn, experimental manipulation of adult neurogenesis in C57Bl/6J mice showed that both the number of Ki67-positive proliferating cells and DCX-positive cells of neuronal lineage decline exponentially, without signs of an intermittent plateau, by 30–40% each month during the first 9 months of the life of mice [213].

Overall, the above animal studies confirm that adult hippocampal neurogenesis is present in the old rodent brain and decreases with age but never disappears as observed sometimes in human research. Although none of the animal species is completely similar to a human with respect to neurogenesis in the adult hippocampus, the above rodents are able to facilitate these studies because of their short lifespan, allowing for the growth of a large number of animals in a short period of time. Large animals are definitely more similar in size to humans and live longer, allowing for longitudinal studies. However, the postnatal continuation of neurogenesis was not observed in all large animals. It was observed that neurogenesis is not present in whales, dolphins, and porpoises [214], but continues postnatally in macaques [200]. Nevertheless, the number of BrdU cells decreased in the DG of macaques with aging [215]. Moreover, transcriptomic analysis of dynamics of hippocampal neurogenesis in macaques identified the new immature neuron markers: STMN1 and STMN2 which were positively verified as immature neuron markers in humans [216].

Interestingly, much attention is paid to the preparation of human samples for analysis including shortening postmortem intervals, improving the fixation conditions and procedures for immunostaining, establishing criteria to reliably antibody signal validation and performing unbiased stereological cell counts as well as new technologies analysis of human samples, such as single-cell RNA-seq [195,209,216,217]. However, it is important to keep in mind that various factors, such as developmental regulators, genetic background, age, disease, nutrient status, and exposure to drugs, hormones, and environmental chemicals also affect neurogenesis in the adult hippocampus [218]. All these parameters are very easily controlled in an animal research experiment. Unfortunately, we cannot control all these parameters in human research. In this context, it will be very difficult to find an indisputable answer to the question of how neurogenesis looks in the adult human hippocampus.

The important breakthrough in obtaining reliable models of human adult hippocampal neurogenesis arose from the successful generation in vitro of pluripotent stem cell-based human hippocampal tissue in early studies from the Fred Gage group in the USA [219] and the Yoshiki Sasai group in Japan [220]. The research of Gage’s group has proved that differentiation in vitro of human ESCs line and patient-derived iPSCs can recapitulate the expression patterns of key developmental genes (e.g., *PROX1*, *NEUROD1* and *TBR1*) typical for hippocampal neurogenesis. Neuronal activity of hippocampal granule neurons obtained in the culture and spontaneous neurotransmitter release were confirmed by whole-cell patch-clamp recordings and Fluo-4AM based calcium imaging respectively [219]. The modeling of hippocampal neurogenesis was upgraded to 3D settings by Yoshiki Sasai’s group. The authors successfully generated dorsomedial telencephalic tissues including the choroid plexus and the hippocampus from hESCs [220] by modulating BMP4 and Wnt signaling. They have shown that obtained hippocampal-like neurons (hippocampal granule neurons and CA3 pyramidal neurons) express typical hippocampal markers (e.g., Prox1 and Zbtb20 expression) and are functional, as revealed by electrophysiology and calcium imaging.

Further possibilities to study hippocampal neurogenesis in vitro stem from great progress in brain organoid field derivation and in vitro culture of human brain region specific organoids (including hippocampal structure) from ESC or iPSC [221,222,223]. Thus the use of human iPSC-derived brain organoids can not only help us better understand the mechanisms underlying the regulation of adult hippocampal neurogenesis, but also provides a new tool to study differences in donor phenotypes based on the transcriptional responses of genes and the time and context-specific epigenomic profiles at various developmental stages. In that respect, the advent of the human brain organoid technology can help us understand human adult neurogenesis.

Finally, it is worth noting that the different brain sizes of rodents and humans can be also one of the most important limitations of adult neurogenesis and could have evolutionarily determined the preference for structural plasticity of the brain over neurogenesis with increasing brain size [224]. In addition, it will be difficult to eliminate, without indisputable specific markers of newborn neurons, the research hypothesis that a slow, delayed maturation of “immature neurons” (INs) from “a reservoir of young neurons” might replace neurogenic processes in the mature brain of human [225]. Nevertheless, from a therapeutic viewpoint, it is crucial to ensure not just the stimulation of adult-born neurons in the hippocampus, but also the control of the different stages of adult hippocampal neurogenesis. The latter can be possible through the use of antidepressants and mesenchymal stem cells. However, the therapeutic strategy based on boosting neurogenesis in the adult human hippocampus is not possible without the strong support of well-controlled animal research experiments and organoid-based studies which allow for better insight into the modification of underlying molecular mechanisms by antidepressants and mesenchymal stem cells.

## Data Availability

Not applicable.
